# Olfactory Dysfunction and Neurotransmitter Disturbance in Olfactory Bulb of Transgenic Mice Expressing Human A53T Mutant α-Synuclein

**DOI:** 10.1371/journal.pone.0119928

**Published:** 2015-03-23

**Authors:** Sufang Zhang, Qian Xiao, Weidong Le

**Affiliations:** 1 Institute of Neurology, RuiJin Hospital, Shanghai Jiao Tong University School of Medicine, Shanghai, China; 2 State Key Laboratory of Stem Cell Biology, Institute of Health Sciences, Shanghai Institutes for Biological Sciences, Chinese Academy of Sciences and Shanghai Jiao Tong University School of Medicine, Shanghai, China; Emory University, UNITED STATES

## Abstract

Parkinson disease is a multi-system neurodegenerative disease characterized by both motor and non-motor symptoms. Hyposmia is one of the early non-motor symptoms occurring in more than 90% of Parkinson disease cases, which can precede motor symptoms even several years. Up to now, the relationship between hyposmia and Parkinson disease remains elusive. Lack of proper animal models of hyposmia restricts the investigation. In this study we assessed olfactory function in Prp-A53T-α-synuclein transgenic (αSyn^A53T^) mice which had been reported to show age-dependent motor impairments and intracytoplasmic inclusions. We also examined cholinergic and dopaminergic systems in olfactory bulb of αSyn^A53T^ mice by immunofluorescent staining, enzyme linked immunosorbent assay and western blot. We found that compared to wild type littermates, αSyn^A53T^ mice at 6 months or older displayed a deficit of odor discrimination and odor detection. No significant changes were found in olfactory memory and odor habituation. Furthermore compared to wildtype littermates, in olfactory bulb of αSyn^A53T^ mice at 10 months old we detected a marked decrease of cholinergic neurons in mitral cell layer and a decrease of acetylcholinesterase activity, while dopaminergic neurons were found increased in glomerular layer, accompanied with an increase of tyrosine hydroxylase protein. Our studies indicate that αSyn^A53T^ mice have olfactory dysfunction before motor deficits occur, and the cholinergic and dopaminergic disturbance might be responsible for the Parkinson disease-related olfactory dysfunction.

## Introduction

Parkinson’s disease (PD) is one of the most popular neurodegenerative disease with approximately 1–2% of the population over 65 years suffering from this disease [1]. It is clinically manifested by motor symptoms and non-motor symptoms, with progressive loss of dopaminergic (DAergic) neurons in substantia nigra (SN) and formation of Lewy bodies (LBs) in surviving cells as its hallmarks [2,3]. It was firstly reported by Ansari and Jonson in 1975 that PD patients might suffer from impairment of odor detection compared to patients of other neurologic diseases such as stroke, epilepsy and cranial injury [4]. Later Ward et al. described that PD patients were defective in both odor detection threshold and discrimination [5]. Multiple studies on olfaction in PD have documented that hyposmia is prevalent in PD patients and it could precede motor symptoms several years [6–10]. However studies on the mechanisms of hyposmia in PD did not draw a coherent view how olfactory dysfunction is affected in PD [11,12]. The paucity of ideal animal models which could mimic olfaction deficiency at the early stage of the disease imposes restriction on the explorations into hyposmia in PD.

It is known that α-synuclein (αSyn) is the main composition of LBs [13]. Various point mutations (A53T, A30P and E46K) in the gene coding αSyn have been found to result in dominant familial PD [2,14–16]. It seems that mutant αSyn has an increased propensity to form filamous inclusion bodies than wt αSyn [17]. Accordingly, Giasson and colleagues generated transgenic (tg) mice overexpressing human A53T αSyn under the mouse prion protein (PrP) gene promoter, and documented that these mice developed age-dependent motor deficits and intracellular αSyn inclusion bodies [17]. In addition to the fibrillar form of αSyn, which is mostly detected in LBs, other forms of αSyn such as oligomers and protofibrils could also possibly contribute to the toxicity of αSyn [18]. Based on these, we assumed that the prefibrillar forms of αSyn possibly participated in the early non-motor symptoms such as hyposmia. Here we took advantage of this tg mouse model to evaluate if the mice carrying human A53TαSyn could manifest hyposmia similar to early PD and then to further explore its possible mechanisms.

In our study, we first evaluated motor function and DAergic neurons in SN of tg mice and wild type (wt) littermates at 10 months (m) old. Then we assessed olfactory functions of tg mice and their littermates at younger age from 3 m old to 10 m old. We found that tg mice displayed a deficit of odor discrimination and odor detection at 6 m old. Based on these, we further examined the pathology of olfactory bulb (OB) and documented a marked decrease of cholinergic neurons in mitral cell layer and an increase of DAergic neurons in glomerular layer of 10-m-old tg mice. Our studies indicate that αSyn^A53T^ mice have olfactory dysfunction before motor deficits and could be used as a research model for hyposmia.

## Materials and Methods

### Ethics Statements

Our experiments were approved by the Experimental Animal Center at Shanghai Jiao Tong University School of Medicine. All procedures in our experiments were performed strictly in accordance with the guidelines of the National Institutes of Health (NIH) for animal care. Every effort was made to minimize suffering and number of animals used. Chloral hydrate was used to anesthetize the animals.

### Animals

Prp-αSyn^A53T^ tg mice were bought from Jackson’s Lab (J004479) and maintained at the Experimental Animal Center at Shanghai Jiao Tong University School of Medicine, in accordance with special pathogen free (SPF) standards. Mice were maintained on a B6/C3H background. All animals were housed in their home cages and maintained on a reverse day/night cycle by artificially changing the light of the room. All tests were performed at the night cycle of animals in an undisturbed room. Standard rodent food and water were available ad libitum except specific experiment period. Room temperature was maintained at 22±1°C, and relative humidity was set at 50±10%. At 1 month, the offspring were genotyped by semi-quantitative polymerase chain reaction (PCR) assay of DNA extracted from tails to discriminate tg mice from wt ones. Then these heterozygous tg mice were crossed to generate homozygous tg mice and quantitative PCR was used to differentiate homozygotes from heterozygotes. We selected male homozygous tg mice and wt littermates for our experiment. At 3 m old, 6 m old and 10 m old, tg (n = 30) and wt littermates (n = 30) were tested on olfactory function including social scent discrimination, non-social scent discrimination, odor memory and odor detection. A rater blind to genotype measured the sniffing time of each mouse and the latency to find the pellet. At 6 m old, wt (n = 12) and tg (n = 12) mice were sacrificed for cholinergic and DAergic staining (n = 3 per group), acetylcholine esterase (AchE) activity (n = 4 per group) and western blot (n = 5 per group) randomly. At 10 m old, wt (n = 13) and tg (n = 13) mice were sacrificed for cholinergic and DAergic staining (n = 4 per group), AchE activity (n = 4 per group) and western blot (n = 5 per group) randomly. The data were analyzed by an observer who was blind to genotype.

### Locomotor activity

Rotarod task was used to evaluate locomotor activity according to the previous study with small modifications [19]. Rotating speed was started at 4 rpm with an acceleration of 10 rpm/minute (min) and the terminal speed must be no more than 40 rpm. Task was ended when the mouse fell down or continuously ran on the rod more than 5 min. During the training period, mice were trained 3 times a day with an interval of 1 hour (h) for 5 consecutive days (d). After one day off, the mice were tested and the time on the rod was recorded and analyzed.

### Olfactory function

#### Social-scent discrimination

A modified block test was designed for assessing discrimination of social scents according to the previous study [20]. Wooden blocks (1.8 cm× 1.8 cm× 1.8 cm) were separately sealed with 5 g of animal beddings from the home cages of test animals in 50 ml of centrifugal tubes for 24 h, so that the blocks took on the odors from animals respectively. In each trial, the test mouse was exposed to one block with its own odor and another block with another mouse’s odor which was totally unfamiliar for the test mouse. A rater blind to genotype measured the time of each mouse spent on sniffing each block in a 120-second (s) trial with a stopwatch (accurate to 0.01 s).

#### Non-social scent discrimination

As previously described [20,21], two glass plates were placed in the home cage. Scent solutions of cinnamon or paprika (100 ng/ml) were freshly prepared and filtered. 25 μl of scent solution was dropped on a small piece of filter paper and put onto the glass plate for the experiment every time. Each mouse was presented with cinnamon solution on one plate and distilled water (control) on the other plate for five successive 3-min trials, separated by a 15-min interval. At the 6th trial, cinnamon solution was replaced with paprika. The whole test process was videotaped with a camera and the time spent on sniffing water or scent solution was respectively recorded. Sniffing was defined as the animal’s nose located 1 cm or less from the odor. The decrease of sniffing time from trial-1 to trial-5 indicates odor habituation or short-term olfactory memory, while a reinstatement of sniffing when presented with a novel odor is defined as dishabituation or odor discrimination.

#### Odor memory

The odor memory was conducted as follows [21]. Test animals were exposed to the same scent twice for 5 min with an inter-trial interval of 20 min, 60 min, or 100 min. The inter-trial interval and odors used in the experiments (banana, peach, or sweet corn, 1:100 dilution, McCormick, MD, USA) were matched randomly. Each experiment was conducted at least 2 days apart. The relative ratio of sniffing time of trial-2 to trial-1 (trial 2/trial 1) was calculated and analyzed.

#### Odor detection

Buried pellet test (bpt) was carried out to evaluate odor detection, which could reflect olfaction threshold in a sense. It was performed as previously described [22]. Individually housed animals were food-restricted on a diet (0.2 g chow per mouse/24 h) from 2 days prior to test and during the experimental period. Body weight was monitored during the period and maintained at 80%~90% of the original body weight. The bpt was performed for 5 consecutive days and each mouse received one trial per day. A 1-g food pellet was buried approximately 0.5 cm below the surface of a 3-cm-deep bedding in the test cage (45 cm × 24 cm × 20 cm). The location of the food pellet was changed daily at random. In each trial, test mouse was placed in the center of the cage. The latency to dig up and eat the buried food pellet was recorded with a stopwatch. If the mouse did not locate the food pellet within 5 min, it would be removed and recorded as 5 min. The bedding in the test chamber was changed between trials. On the 6th day, a visual pellet test (vpt) was carried out to make sure that the animals did not suffer from altered locomotor activity or motivation. The vpt was conducted in a similar way as the bpt except that the food pellet was placed on the surface of the bedding.

### Immunostaining

Animals were anesthetized with chloral hydrate, and perfused with 30 ml of phosphate buffer solution (PBS, 0.1M, pH7.2) followed by 30 ml of 4% (w/v) paraformaldehyde fixative (phosphate buffered; 0.1 M, pH7.2). Brains were postfixed in paraformaldehyde overnight and dehydrated gradually in 15% (w/v), 30% (w/v) sucrose solutions. Then the tissue was coronally sectioned (8 μm for the OB and 12 μm for the midbrain) using a freezing microtome. For immunostaining, sections were added with 1% (v/v) hydrogen peroxide (H_2_O_2_) to remove endogenous hydrogen peroxidase and then blocked with goat serum for 30 min. After being incubated at 4°C with rabbit anti-tyrosine hydroxylase (TH; 1:800; Millipore, Billerica, MA, USA) antibody overnight, the slices were sequentially incubated with biotinylated secondary antibody (1:200; Westang, Shanghai, PRC) for 1 h and avidin-biotin reagent (Vector Labs, Burlingame, CA) for 1 h and then visualized with DAB (Vector Labs). For immunofluorescent staining, sections were incubated with mouse anti-TH (1:800; Sigma, St. Louis, MO, USA) and rabbit anti-choline acetyltransferase (ChAT; 1:1000; Abcam, Cambridge, UK) antibody at 4°C overnight, and then incubated with secondary antibodies conjugated with Alexa Fluor-488 or 555 (1:400; Invitrogen, Carlsbad, CA, USA) for 1 h and with DAPI for 1–2 min. Sections were viewed and photographed with a fluorescent microscopy (Olympus IX81). For counting DAergic neurons in SN, TH^+^ neurons were counted in every 4 slices from AP-2.7 to AP-4.0 mm and the sum multiplied by 4 was taken as the total number of TH^+^ neurons in SN. For counting neurons in OB, the positive neurons were counted in every 6 slices from AP 4.2 to AP 3.2 mm and the average number was analyzed. For statistical analysis, at least 3 wt and 3 tg mice were counted. The number of ChAT^+^ neurons in OB were counted in the view of 10×objective and the TH^+^ neurons in OB were counted in the view of 20×objective. Cell counting was conducted by an observer who was blind to the genotyping.

### Western blot

Different tissues of brains from wt and tg mice were weighed and homogenized in RIPA (10 μl/ mg tissue) plus 1% (v/v) PMSF on ice. Protein concentration was measured with the BCA Protein Assay Kit (Pierce, Rockford, IL, USA). Thirty mg of total protein per sample was loaded to 12.5% (w/v) acrylamide gels and after electrophoresis transferred to PVDF membranes. These membranes were blocked with 5% (w/v) skim milk in TBST for 1 h at room temperature, and then incubated with mouse anti-TH antibody (1:2000; Sigma), mouse anti-αSyn (1:3000; Abcam) or rabbit anti-phosphorylated (ser129) αSyn (p-αSyn, 1:5000; Abcam) overnight at 4°C. After extensive washing, the membranes were incubated with horseradish peroxidase (HRP) conjugated anti-mouse or anti-rabbit secondary antibody (1:2000; Cell Signaling, Danvers, MA, USA) for 2 h at room temperature and detected with the SuperSignal Detection Kit (Pierce).

### AchE activity

AchE activity was measured in a colorimetric method with the AchE activity kit (Jiancheng Bioengineering Institute, Nanjing, PRC). First, the OB and frontal cortex were weighed and homogenized in 0.9% (w/v) sodium chloride solution (9 μL/mg tissue). Then samples were centrifuged at 2500 g at 4°C and the supernatant was collected for measurement. After that, the samples were incubated with a reaction mixture that contained acetylcholine and sulfhydryl chromogenic agent at 37°C for 6 min. Then the reaction was ended and optical density (OD) was measured at 412 nm with a microplate reader. One unit of AchE activity was defined as the number of micromoles of acetylcholine hydrolyzed per mg of protein per min.

### Statistical analysis

Results were presented as the mean±SEM. Cell counts between wt and tg mice were analyzed by Student’s t-test. Quantative data of olfactory function, AchE activity and TH proteins were analyzed by two-way ANOVA with Bonferroni’s *post hoc* analysis. Levels of significance were set at 0.05.

## Results

### αSyn^A53T^ mice did not have nigral DAergic neuron loss nor locomotor deficit

**Fig 1:**
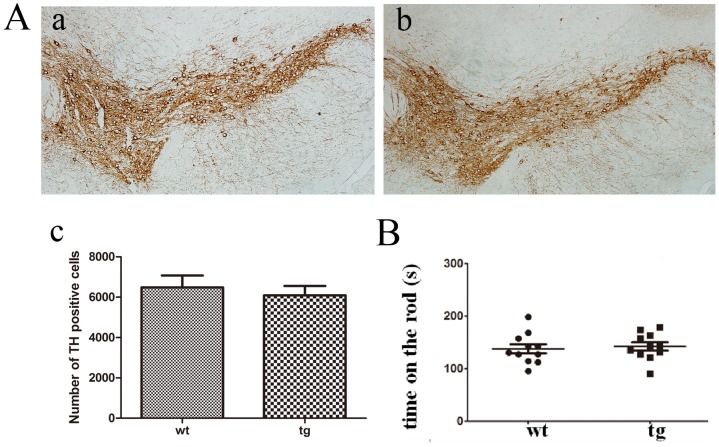
TH staining and locomotor activity. A (a, b, c): TH immunostaining in SN of wt (n = 4, a) and tg (n = 5, b) mice showed no significant difference at 10 m old. B: Time on the rod in the rotarod test showed no difference between tg (n = 11) and their wt littermates (n = 11) at 10 m old. The quantitative data were expressed as mean±SEM and analyzed by Student’s t-test.

**Fig 2:**
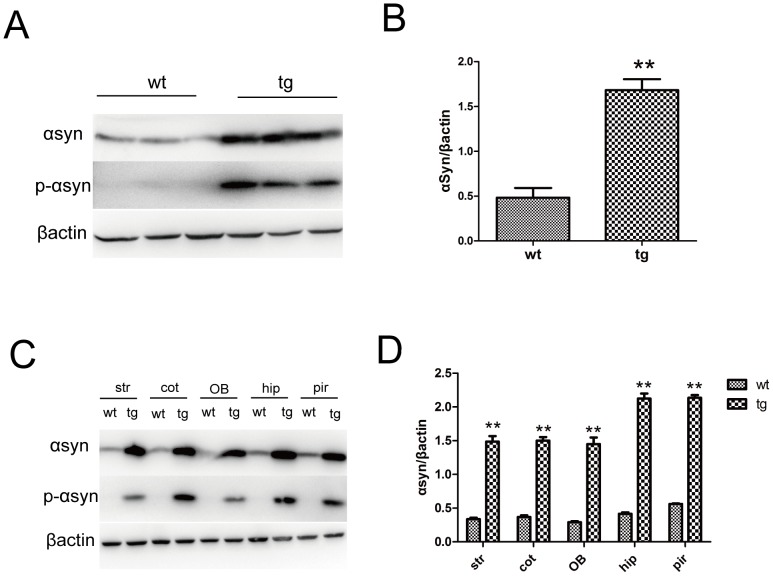
Expression levels of αSyn and phosphorylated αSyn (ser129) in brains of wt and tg mice at 10 m old. A, B: Tg mice expressed more αSyn and phosphorylated αSyn (p-αSyn) in frontal cortex than wt mice (n = 3 per group). ** p< 0.01, tg vs wt. The quantitative data were expressed as mean±SEM and analyzed by Student’s t-test. C, D: Compared to wt littermates, tg mice showed much higher αSyn and p-αSyn in different tissues of brain including the striatum, frontal cortex, OB, hippocampus and piriform cortex. ** p<0.01, tg vs wt. The quantitative data were expressed as mean±SEM and analyzed by two-way ANOVA. Str: striatum; cot: frontal cortex; OB, olfactory bulb; hip: hippocampus; pir: piriform cortex.

The morphology and number of TH^+^ neurons in SN showed no difference between wt and tg mice at 10 m old (Fig. 1A). There was also no difference of rotarod performance between the two groups, indicating that locomotor function is not obviously affected in 10-m-old tg mice (Fig. 1B). Therefore, we consider that tg mice below 10 m old to be asymptomatic of motor dysfunction. We also measured the expression levels of αSyn and p-αSyn in different tissues of brains from 10-m-old wt and tg mice by western blot and found tg mice expressed high levels of αSyn and p-αSyn throughout the brain including OB and piriform cortex (Fig. 2)

### αSyn^A53T^ mice showed olfactory impairment

**Fig 3:**
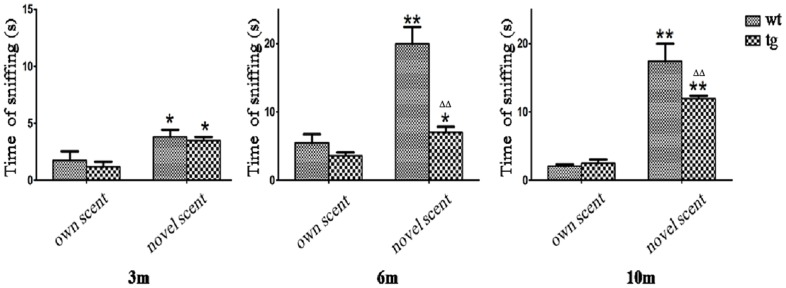
Social scent discrimination. Both wt and tg mice spent more time on a block with other mouse’s scent than the block carrying its own scent. 3 m old: wt n = 3, tg n = 3; 6 m old: wt n = 3, tg n = 3; 10 m old: wt n = 3, tg n = 4. * p<0.05, ** p<0.01, time on novel scent vs own scent. ΔΔ p< 0.01, tg vs wt on novel scent. The quantitative data were expressed as mean±SEM and analyzed by two-way ANOVA.

In the social scent discrimination task, tg mice seemed to sniff the novel scent less than wt mice at 6 m and 10 m old, but as wt littermates did, tg mice of all ages (3 m, 6 m and 10 m old) spent more time sniffing the novel block than the block which took on its own scent, which reflects a relatively normal function in the social scent discrimination (Fig. 3).

**Fig 4:**
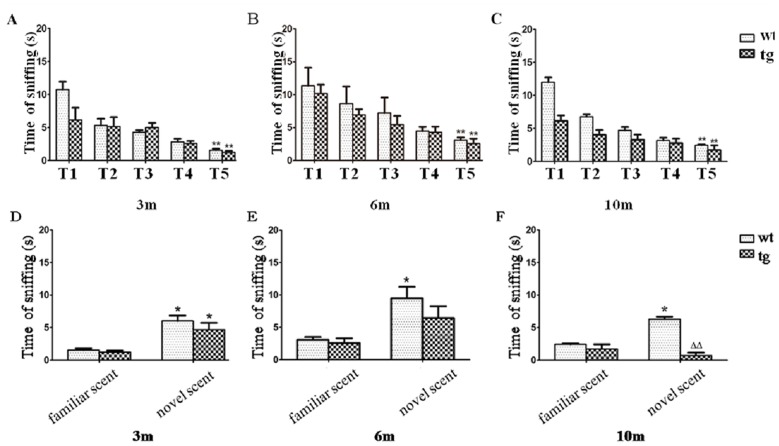
Odor habituation and discrimination on a non-social scent. A, B and C: As trials progressed, all mice spent gradually reduced time on contacting with a non-social odor. 3 m old: wt n = 3, tg n = 3; 6 m old: wt n = 4, tg n = 4; 10 m old: wt n = 3, tg n = 4. ** p<0.01, trial-5 (T5) vs trial-1 (T1). No significant difference of contacting time in trial-5 was present between wt and tg mice. D, E and F: At 3 m old, both wt and tg mice spent more time on a novel scent (trial-6) than the familiar scent (trial-5). However, at 6 m and 10 m old, tg mice did not sniff the novel scent (trial-6) more than the familiar scent (trial-5). * p< 0.05, time on novel scent vs familiar scent. ΔΔ p< 0.01, tg vs wt on novel scent. The quantitative data were expressed as mean±SEM and analyzed by two-way ANOVA.

In the non-social scent discrimination task, the sniffing time of wt and tg mice both decreased from trial-1 to trial-5, which indicates normal odor habituation or short-term olfactory memory. No significant difference of contacting time in trial-5 was recorded between wt and tg mice (Fig. 4). At 3 m old, both wt and tg mice spent more time on the new odor than the odor they had been familiarized, indicating normal dishabituation and non-social odor discrimination. There was no significant difference in the contacting time of trial-6 (novel scent) between wt and tg mice. However at 6 and 10 m old, tg mice did not spend more time sniffing the novel scent (trial-6) than the familiar scent (trial-5) as wt mice did, implying an impaired non-social scent discrimination in tg mice (Fig. 4).

**Fig 5:**
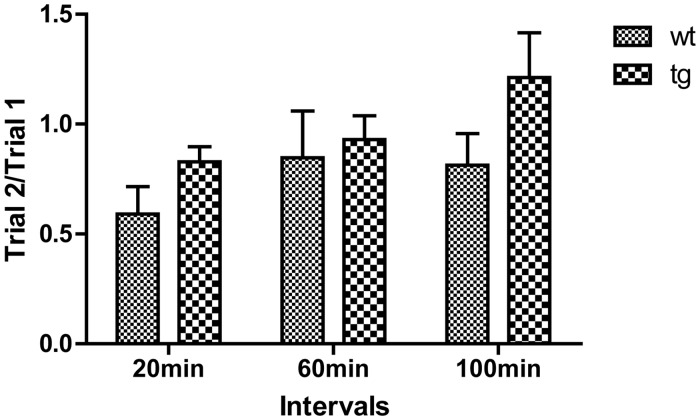
Odor memory test. Tg mice at 10 m old seem to spend slightly more time than age-matched wt mice at all intervals on an odor encountered before, but the statistical analysis showed no significant difference between tg (n = 16) and wt (n = 9) controls. The ratios of trial 2/trial 1 were squared, expressed as mean±SEM and analyzed by two-way ANOVA.

In the odor memory test, the ratio of trial-2 to trial-1(trial 2/trial 1) was used to evaluate odor memory. When inter-trial intervals were set at 20 or 60 min, both wt and tg mice spent less time sniffing the previously encountered odor. However, when the trial interval was increased to 100 min, unlike wt mice tg mice sniffed the odor at the second exposure more than the first exposure, with the average trial 2/trial 1 more than 1. Although the difference was not statistically significant, tg mice tended to have worse olfactory memory performance than wt mice at 10 m old (Fig. 5).

**Fig 6:**
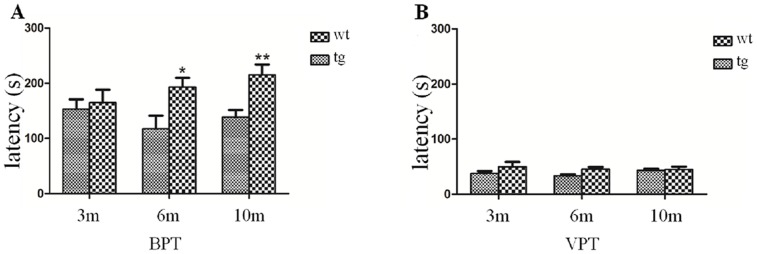
Performance in the odor detection task. A: In the buried pellet test (bpt), tg mice at 6 m old showed a longer latency to find the pellet than wt mice, and the time got much longer when they grew up to 10 m old. 3 m old: wt n = 15, tg n = 15; 6 m old: wt n = 15, tg n = 15; 10 m old: wt n = 10, tg n = 17. * p< 0.05, ** p< 0.01, tg vs wt. B: All the animals performed well in the visual pellet test (vpt). No significant difference was found in the visual pellet test between wt and tg mice. 3 m old: wt n = 15, tg n = 15; 6 m old: wt n = 15, tg n = 15; 10 m old: wt n = 10, tg n = 17. The quantitative data were expressed as mean±SEM and analyzed by two-way ANOVA.

In the bpt, the latency to find the pellet did not differ significantly between wt and tg mice at 3 m old, whereas at 6 m and 10 m old more time was needed for tg mice to locate the buried food than wt mice, indicating an impaired odor detection (Fig. 6A). In the vpt which was set to exclude the possible influence of motor function or motivation, the two groups behaved similarly (Fig. 6B).

### Decreased cholinergic neurons and cholinergic activity in OB of αSyn^A53T^ mice

**Fig 7:**
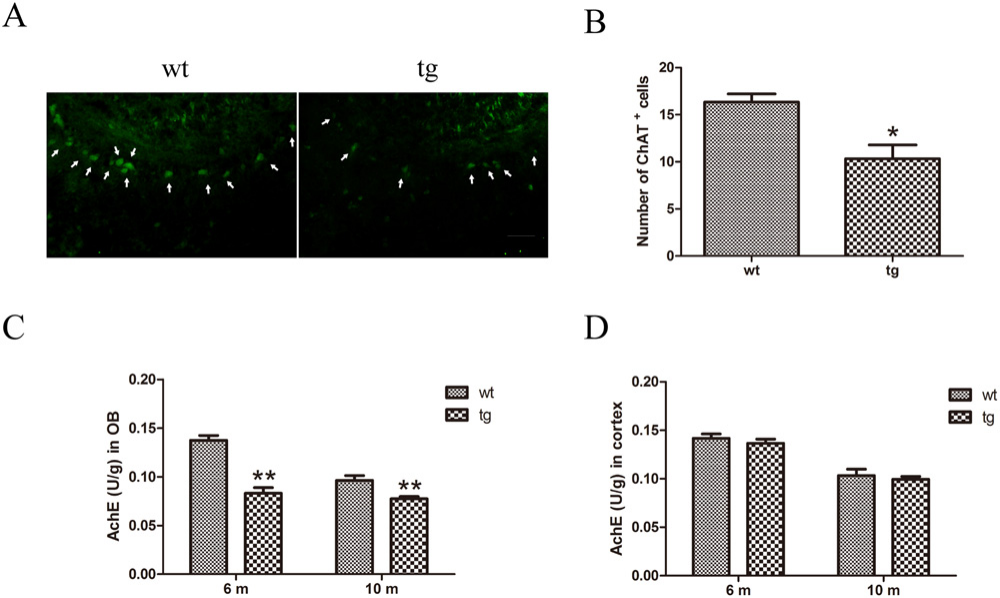
Cholinergic denervation in OB of tg mice. A and B: ChAT^+^ cells were decreased in mitral cell layer of OB in tg mice (n = 4) at 10 m old than wt littermates (n = 4). * p<0.05 tg vs wt. Scale bar: 100 μm. Arrows indicate the ChAT^+^ cells. The quantitative data were expressed as mean±SEM and were analyzed by Student’s t-test. C: AchE activity in OB of tg mice (n = 4 in each age group) declined from 6 m old compared with wt mice (n = 4 in each age group). ** p<0.01, tg vs wt. The quantitative data were expressed as mean±SEM and were analyzed by two-way ANOVA. D: In cerebral cortex, AchE activity did not change between tg (n = 4 in each age group) and wt controls (n = 4 in each age group) at 10 m old. The quantitative data were expressed as mean±SEM and analyzed by two-way ANOVA.

Cholinergic neurons were detected with immunofluorescent staining of ChAT. Compared with wt mice, the number of cholinergic neurons in tg mice showed a mild trend to decrease at 6 m old, yet no significant difference existed between wt and tg mice (S1 Fig.). At 10 m old a significant decline of ChAT^+^ neurons in mitral layer of OB was found in tg mice compared with wt littermates (Fig. 7A, B).

AchE activity of OB in wt and tg mice was measured by enzyme linked immunosorbent assay (ELISA). Significantly lower AchE activity was found in tg mice than wt mice at 6 and 10 m old, whereas no difference was found in cortex before 10 m old, implying that in tg mice decline of AchE activity in OB precedes that in cortex (Fig. 7C, D).

### Increased DAergic neurons and TH protein levels in OB of αSyn^A53T^ mice

**Fig 8:**
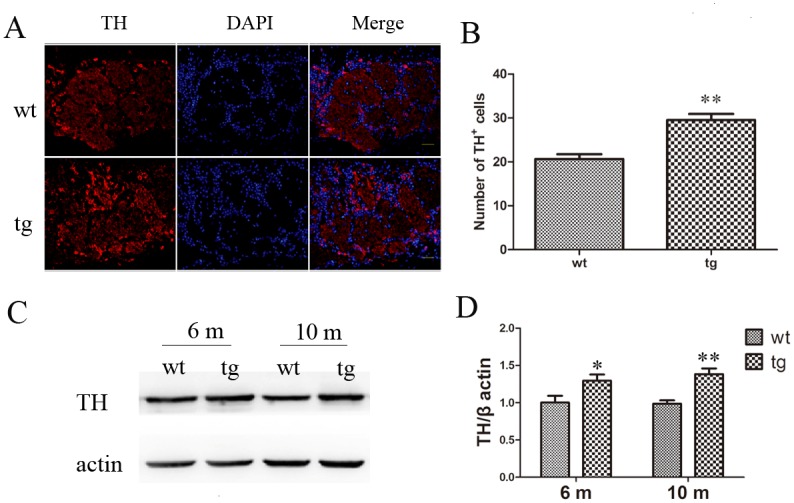
Increased DAergic neurons and TH level in OB of tg mice. A and B: TH^+^ neurons were increased in glomerular layer of OB in tg mice (n = 4) at 10 m old than wt littermates (n = 4). ** p<0.01, tg vs wt. Scale bar: 30 μm. Arrows indicate the TH^+^ cells. The quantitative data were expressed as mean±SEM and analyzed by Student’s t-test. C and D: Higher TH protein level was found in OB of tg mice (n = 5 in each age group) after 6 m old than wt mice (n = 5 in each age group). * p< 0.05, ** p< 0.01, tg vs wt. The quantitative data were expressed as mean±SEM and analyzed by two-way ANOVA.

Immunofluorescent staining provides a credit evidence of DAergic alteration in OB. We found an increased number of TH^+^ neurons in glomerular layer of 10-m-old tg mice than wt littermates (Fig. 8A, B). Consistent with this, TH protein in OB of tg mice was moderately increased at 10 m old (Fig. 8C, D). Although TH protein in OB of 6-m-old tg mice already had a moderate increase, tg mice at 6 m old did not show significantly more DAergic neurons in OB than wt littermates, suggesting functional changes may occur at 6 m old, prior to the morphological change of the neurons (S1 Fig.).

## Discussion

In this study we found that αSyn^A53T^ mice at 10 m old displayed partial olfactory dysfunction without any motor deficit and nigral DAergic neuron loss, which resembles the observation that olfactory dysfunction usually precedes motor deficits in PD patients [8–10]. Hereby we believe that the αSyn^A53T^ mice could be a proper rodent model to mimic olfactory dysfunction at the early stage of PD. Based on this animal model, we further documented the cholinergic and DAergic disturbance in OB of αSyn^A53T^ mice.

### Olfactory dysfunction in mice expressing human αSyn^A53T^

Olfactory function could be evaluated from different domains including odor identification, discrimination, odor threshold and odor memory [23]. Different from humans, rodents have two olfactory pathways: main olfactory system and accessory olfactory system, both of which are involved in the detection of odors. Although the roles of the two systems seem to be partly overlapping, the main olfactory system has been generally thought to detect common odors in the environment and accessory olfactory system plays a main role in pheromones perception [24]. Based on this, odor discrimination comprises of social scent and non-social scent discrimination. In our study we found that αSyn^A53T^ mice at 6 m old exhibited a subtle deficiency in distinguishing non-social odor, which was gradually worse with ages. However, in the block test designed to evaluate the social scent discrimination, both wt and tg mice spent much more time on other mouse’s scent compared to their own. The social and non-social discrimination tests suggest that at the early stage of the disease, the main olfactory system might be predominantly dysfunctional and the accessory OB system might be spared.

Odor detection reflects olfactory threshold. Alteration of threshold was the first olfactory deficits identified in PD patients [4]. Hummel et al found odor thresholds declined most dramatically with age compared to odor identification and odor discrimination [25]. Multiple odors could be used to test olfactory threshold, in which ascending or descending concentrations of odors are presented to the subject in order to find the lowest concentration the subject could detect [23]. In our study, we used a much simpler and also quite common method to test the olfactory threshold. We found that αSyn^A53T^ mice at 6 m old already showed reduced ability to locate the hidden pellet, implying that the alteration of threshold was among the earliest olfactory deficits in this model. It is unlikely that the declined ability is attributed to lack of motivation or impaired motor function, because tg mice spent the same time to find the visual pellet as wt mice did.

Odor memory consists of short-term memory and long-term memory. Distinct pathways are involved in short-term memory and long-term memory. Generally, short-term memory formation needs an extensive neural network, in which the entorhinal cortex plays a pivotal role, whereas once memory consolidates a limited working network (the OB and piriform cortex) is needed for long-term memory recall [26]. In our study we examined the short-term memory which demanded more extensive neural function. In the non-social scent discrimination test, the gradual decline of trial-5 to trial-1 indicates a normal short-term memory at 5-min delay. In the olfactory memory test, when trial interval was set to be 20 min or 60 min, both wt and tg mice could recognize the previously encountered scent and the average trial 2/trial 1 was below 1, indicating that the memory formation in αSyn^A53T^ mice was not impaired and the scent information could be maintained for at least 60 min at 10-m-old wt and tg mice. When trial interval was increased to 100 min, the average trial 2/trial 1 of tg mice increased to be more than 1, while the average ratio of wt mice still remained below 1. However the difference was not statistically significant, which we consider could be ascribed to the limited sample size, or insufficient sensitivity of the method.

### Mice expressing human αSyn^A53T^ might mimic early sign of PD

It is generally recognized that when PD patients develop motor symptoms, at least 60% of DAergic neurons in SN have been lost [2]. Before this advanced period, a large range of non-motor symptoms have already appeared, among which hyposmia is one of the earliest symptoms in PD [3]. According to Braak’s staging of PD, OB is also one of the earliest involved regions in stage 1 [27,28]. Intriguingly, olfactory tract could bypass blood-brain barrier and project to multiple central structures [29]. Based on these, some researchers believe that olfactory dysfunction is not only an early sign of PD, but also a potential culprit behind PD [11,12]. So far it still remains elusive whether the olfactory system is the initiation site of PD pathology. Lack of proper animal models limits the relevant researches at this point. To address this question, numerous models based on genetic mutations have been generated, but few reports about hyposmia in these models have been presented.

Our findings in αSyn^A53T^ mice are of interest to detect the early sign and progression of PD. These αSyn^A53T^ mice at 10 m old did not display motor dysfunction and obvious DAergic neuron degeneration, although they expressed 3–5 fold higher αSyn and much higher p-αSyn (ser129) throughout the brain than wt mice. The homozygous Prp-αSyn^A53T^ tg mice were reported in Giasson’s study to develop motor impairments from 8 m old [17]. However in our lab, the homozygous Prp-αSyn^A53T^ tg mice behaved normally in the rotarod test at 10 m old. In another study, homozygous Prp-αSyn^A53T^ tg mice and wt mice behaved no differently in the ratorod test at 12 m old, which is in accordance with our data [30]. Instead, these mice showed olfactory deficits, implying that the overexpression of mutant αSyn could lead to olfactory dysfunction and olfactory system could be more vulnerable to the toxicity of mutant αSyn. Consistently, partial olfactory deficits have been reported in wt αSyn tg mice under the Thy1 promoter or mouse αSyn promoter [31,32], providing evidence that either mutant αSyn or wt αSyn is sufficient to result in olfactory impairments. However, in their study, mice transgenic for wt αSyn under the control of mouse αSyn promoter showed some olfactory dysfunction at 10–11 m old. The authors did not detect younger mice [32]. Our research provided detailed and comprehensive evaluation of olfactory function from 3 m to 10 m old. Mice overexpressing wt αSyn under the Thy1 promoter did not show obvious motor deficits [31]. Differently, αSyn^A53T^ mice used in our study exhibited age-dependent motor symptoms and pre-motor symptoms accompanied by high level of αSyn in OB and other brain regions, which may better assembly the features of human PD. More importantly, we performed neuropathology and found a significant changes of cholinergic and DAergic neurons in the olfactory bulb. Therefore we think the Prp-A53T-α-synuclein mice in our study are suitable for the future researches of pre-motor olfactory dysfunction. However, other study yielded contradictory result that no profound olfactory dysfunction was seen in mice transgenic for artificial chromosomes containing A53T αSyn at 12 m old [33]. We believe this inconsistency could be due to different tg mice and olfaction assessment methods. For example, in the latter study, neither odor discrimination nor odor memory was evaluated and their mouse line did not exhibit any αSyn aggregations even at 22 m old [33].

### Alteration of cholinergic and DAergic system in OB may be related to hyposmia in αSyn^A53T^ mice

The mechanisms of hyposmia in PD are not clear yet. Olfactory nerve atrophy, loss of olfactory neurons, formation of Lewy body in olfactory system [34], alteration of neural transmitters [35–37], decreased olfactory neurogenesis [38], declination of metabolism in piriform cortex and amygdale [39], and atrophy in limbic and paralimbic cortex [40] have been suggested to be associated with hyposmia. Hyposmia has been reported to be irrelevant to disease severity, duration and levodopa administration [7,41,42]. It would not get worsen as PD progresses, at least at the symptomatic period, which raises the question whether other pathophysiological mechanisms than DAergic loss in striatum contribute to hyposmia in PD. Increasing evidences highlight the importance of cholinergic loss in pathophysiological changes of non-motor symptoms in PD [43]. The nucleus basalis of Meynert in basal forebrain projects the majority of cholinergic input to cerebral cortex [44]. This nucleus was shown to undergo severe degeneration in patients of PD [45]. In a positron emission tomography (PET) study, a positive association between limbic AchE activity and olfactory function was documented [37]. Shimada et al used PET to measure brain AchE activity in PD patients and found AchE activity was significantly decreased in the cerebral cortex, whereas there was no difference between the early PD and advanced PD in the extent of reduction, indicating that the cholinergic system defect starts early in PD [46]. This phenomenon is compatible with the observation that hyposmia occurs early and does not get worse as the disease advances [7,46]. Animal studies also provided evidences for the involvement of cholinergic system in olfactory processing. For example, it has been reported that disruption of cholinergic system with acetylcholine receptor antagonist can cause impaired odor memory and odor discrimination [47–50], which could be alleviated by AchE inhibitor [49]. Administration of AchE inhibitor could enhance odor discrimination performance of normal rats in a complex odor discrimination task [51]. However, there is little known about the cholinergic alteration in OB in PD patients or animal models. In our study we found cholinergic neurons existed in mitral cell layer of OB and decreased in olfaction-impaired tg mice. For a long period, the existence of intrinsic cholinergic interneurons in OB remains controversial. In a recent study, Krosnowski et al. took advantage of the ChAT^(BAC)^-eGFP tg mice to visualize ChAT^+^ cells and found a large number of cholinergic neurons in OB, which resided in all layers of OB, suggesting that OB receives not only the modulation of central cholinergic projection, but also the local cholinergic networks [52]. Given that axons of mitral cells make up the main output of OB and acetylcholine receptors (AchRs) exist on mitral cells [29,53], it is inferred that cholinergic interneurons in mitral cell layer could possibly play a direct modulation and the changes of these interneurons could influence the olfactory performance.

In our study, we also found that DAergic neurons were moderately increased in glomerular layer of OB in the PD mouse model. A similar increase of periglomerular DAergic neurons has also been reported in other studies with PD post-mortem tissues [54,55] and in animals chronically exposed to 1-methyl-4-phenyl-1,2,3,6-tetrahydropyridine (MPTP) [56]. As the neurotransmitter of periglomerular cells, DA exerts an inhibitory effect on mitral cells, the axons of which constitute the main output of OB [29]. Previous study showed that dopamine receptor 2 (D2) agonist quinpirole decreased the odor detection performance of rats, while pretreatment of D2 antagonist spiperone blocked this effect [57]. Hsia et al. reported that DA receptor activation in OB depressed the synaptic transmission between olfactory receptor neurons and mitral cells [58]. Hereby we infer that the increased DAergic periglomerular neurons might inhibit olfactory transmission and contribute to the declined olfactory function in our experiment.

The mechanisms for the disturbance of cholinergic and DAergic systems in OB of tg mice remain unresolved. We infer that the toxicity of mutant αSyn could possibly play a role. αSyn was reported to share structural and functional homology with 14–3–3 protein and overexpressing mutant αSyn^A53T^ may have more toxicity by binding with 14–3–3 associating proteins than wt αSyn [59]. 14–3–3 protein could also interact directly with neuronal nicotinic AchRs and this interaction was reported to increase the steady-state level of AchRs [60]. Since the nicotinic AchRs exist in the periglomerular interneurons and mitral cells [53], it is possible that mutant αSyn may disturb the function of periglomerular interneurons and mitral cells. Besides, a possible link between the cholinergic system and DAergic system in OB may exist [60]. For example by immunostaining, muscarinic AchRs were found to express in DAergic neurons of OB [61]. Administration of acetylcholine could cause a reduced spontaneous firing of periglomerular DAergic interneurons, which could be blocked by atropine and mimicked by the M2 agonist oxotremorine [62]. Therefore we could not exclude the possibility that the increase of DAergic neurons might be a compensation of the reduced innervation of cholinergic interneurons. Although the function of local cholinergic network remains unclear, it is likely that it could connect into different circuit and play an important regulatory role[52].

Since it remains to be elucidated whether the cholinergic or DAergic neuron disturbance in our mouse model takes the initial and pivotal role in the pathogenesis of hyposmia, further efforts should be made in this model to interfere with cholinergic and DAergic systems respectively to see if the PD-related hyposmia would be altered. The αSyn^A53T^ mice will be useful for further insights into the mechanisms of hyposmia and to screen for potential interventions.

## Supporting Information

S1 Fig Immunostaining of cholinergic and DAergic neurons in OB of 6-m-old mice. A and B: Immunofluorescent staining of ChAT (A) and TH (B) showed no significant difference between wt and tg mice at 6 m old (n = 3 for each group). Scale bar in A: 200μm. Scale bar in B: 50μm. Arrows indicate the ChAT^+^ cells. C and D: Statistical analysis of the number of ChAT^+^ cells (C) and TH^+^ cells (D) in OB found no significant difference between 6-m-old wt and tg mice. The quantitative data were expressed as mean±SEM and analyzed by Student’s t-test.
(TIF)
