# Supplementary figures and images for: Olfactory Dysfunction and Neurotransmitter Disturbance in Olfactory Bulb of Transgenic Mice Expressing Human A53T Mutant α-Synuclein
Source: PLoS One. 2015 Mar 23;10(3):e0119928. doi: 10.1371/journal.pone.0119928 (PMC4370499; doi:10.1371/journal.pone.0119928)

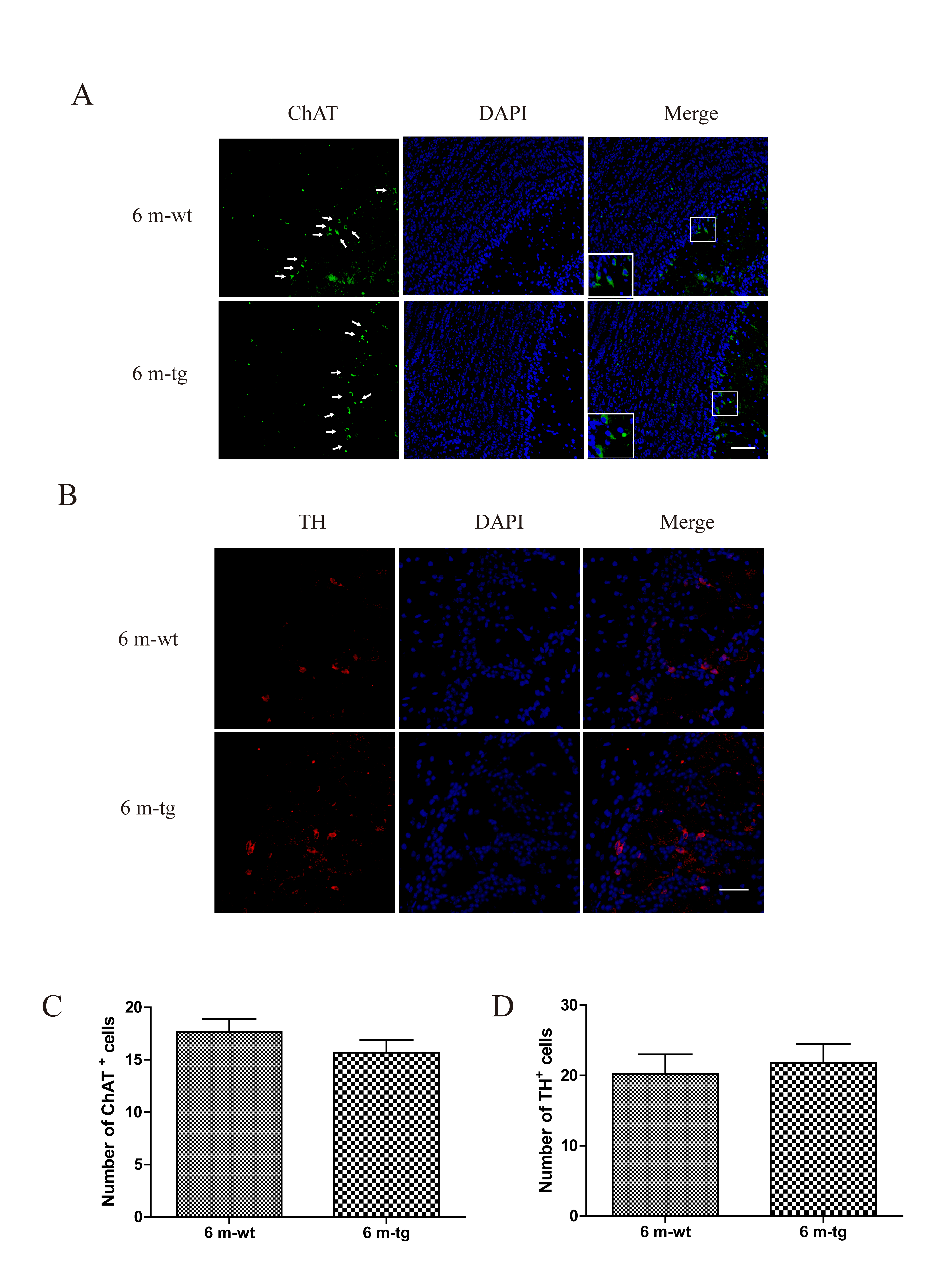

Supplement: S1 Fig — A and B: Immunofluorescent staining of ChAT (A) and TH (B) showed no significant difference between wt and tg mice at 6 m old (n = 3 for each group). Scale bar in A: 200μm. Scale bar in B: 50μm. Arrows indicate the ChAT+ cells. C and D: Statistical analysis of the number of ChAT+ cells (C) and TH+ cells (D) in OB found no significant difference between 6-m-old wt and tg mice. The quantitative data were expressed as mean±SEM and analyzed by Student’s t-test. (TIF) [file pone.0119928.s001.tif]
